# PFKFB3 alleviates the advancement of *Fusarium solani* keratitis by attenuating macrophage inflammation

**DOI:** 10.3389/fcimb.2025.1623027

**Published:** 2025-12-04

**Authors:** Yani Zhang, Yanqing Zhang, Hanfeng Tang, Jianzhang Hu

**Affiliations:** Department of Ophthalmology, Fujian Medical University Union Hospital, Fuzhou, China

**Keywords:** Fungal keratitis, inflammation, PFKFB3, BMDM, glycolysis

## Abstract

**Objective:**

To investigate the anti-inflammatory effect of glycolysis rate-limiting enzyme 6-phosphofructose-2-kinase/fructose-2, 6-biphosphatase 3 (PFKFB3) in fungal keratitis (FK) infected by *Fusarium solani* (*F. solani*).

**Methods:**

We identified the up-regulation of PFKFB3 in fungal keratitis via western blot, quantitative real-time polymerase chain reaction (RT-PCR), and immunofluorescence staining. Subsequently, elucidated the augmentation of glycolytic flux in cornea and bone marrow-derived macrophages (BMDM) following *F. solani* invasion by RT-PCR, cellular energy metabolism analyzer, and lactate content assay. After that, we reduced PFKFB3 expression utilizing small interfering RNA (siRNA) *in vitro* and adeno-associated virus (AAV) *in vivo* and also assessed the expression levels of inflammatory factors. The severity of corneal infection following PFKFB3 depletion was checked by slit-lamp microscopy, corneal OCT, and H&E staining. Ultimately, we assessed the phosphorylation status of the PI3K/AKT/NF-κB p65 signaling pathway following PFKFB3 suppression via western blot and immunofluorescence staining.

**Results:**

PFKFB3 was highly triggered in *F. solani*-infected corneas and BMDM compared to normal tissue. Besides, infection with *F. solani* promotes the increase of inflammatory mediators and glycolytic flux in the cornea and BMDM. Whereas inflammation in BMDM and the degree of fungal keratitis lesions worsen by suppressing PFKFB3 expression, which increased corneal ulcer infiltration, elevated clinical scores, enhanced corneal thickness, and upregulation of inflammatory signals could be demonstrated. Furthermore, we found that *F. solani* infection can activate the phosphorylation of PI3K/AKT/NF-κB p65 at low PFKFB3 expression levels.

**Conclusions:**

In *F. solani*-infected corneas and BMDM, the glycolysis rate-limiting enzyme PFKFB3 was markedly upregulated. After infection, moderate PFKFB3 activation effectively mitigates inflammation and the progression of fungal keratitis. Moreover, activated PFKFB3 may rely on the PI3K/AKT/NF-κB p65 signaling pathway to safeguard the cornea from further damage due to inflammation.

## 1 Introduction

Fungal keratitis (FK) is a prevalent cause of corneal blindness, typically occurring in tropical and subtropical regions where agricultural practices are widespread (Hoffman et al., 2021). It is marked by blurred vision, ocular pain, heightened discharge, and lacrimation, and if neglected, it may advance to deep ulcers, posterior elastic lamina bulging, accumulation of pus in the anterior chamber, and even endophthalmitis (Florcruz and Peczon, 2008; Brown et al., 2021). Fusarium, Candida yeast, and Aspergillus comprise 95% of the species for FK infections (Brown et al., 2021). The primary treatment for fungal keratitis involves a combination of pharmacological agents and corneal transplantation; nevertheless, the treatment poses challenges due to medication resistance, inadequate penetration, and the intricacies of the surgical procedure (Huang et al., 2016).

Macrophages are essential immune system components, heavily contributing to regulating inflammatory responses, eliminating pathogens, and the overall immunological response (Yu et al., 2024, 2023; Li et al., 2023). Macrophages are categorized based on their activation status into the classical activated state M1 and the selectively activated state M2 (Song et al., 2022). Macrophages primarily participate in pro-inflammatory reactions, characterized by markers such as interleukin (IL)-1β, IL-12, and tumor necrosis factor-alpha (TNF-α) (Wang et al., 2019). Macrophages, as the most rapidly recruited immune cells to infection sites, are directly implicated in antifungal defense and are crucial for the phagocytosis and destruction of fungal spores (Ghosh et al., 2023; Lin et al., 2017). External stimulus to the cornea, like trauma or microbial infection, can cause significant macrophage buildup at the limbus and central cornea (Hu et al., 2014). Macrophages serve a dual role in fungal clearance; upon recognizing fungal pathogens, they eliminate them by recruiting neutrophils, augmenting oxidative stress, and boosting the immune response, besides provoking an inflammation reaction and incurring tissue damage (Yu et al., 2024).

6-phosphofructo-2-kinase/fructose-2,6-bisphosphatase 3 (PFKFB3) is an isoenzyme of phosphofructokinase-2 (PFK2) that helps the synthesis of fructose-2,6-bisphosphate (F-2,6-P2), which allosterically activates PFK1, hence enhancing glycolytic fluxes (Min et al., 2021). Among the PFKFB isozymes, PFKFB3, a bifunctional enzyme, shows the highest kinase to bisphosphatase activity, thus promoting the preservation of intracellular F-2,6-P2 levels (De Bock et al., 2013). PFKFB3 has been implicated with diabetes and its complications, malignancies, inflammatory illnesses, and neovascular ocular conditions (Min et al., 2021; Zhou et al., 2022; Liu P, et al., 2024; Liu et al., 2020). The regulation of PFKFB3-mediated glycolysis and inflammatory illnesses has been extensively researched in recent years; however, the regulatory mechanisms vary across various ailments and cell types. Chen et al. established that PFKFB3, which is strongly expressed in aortitis, is involved in macrophage M1 polarization (Chen et al., 2022). In ulcerative colitis, the hypoxia-inducible factor HIF-1α supports neutrophil survival and functional maintenance at the inflammatory site by driving PFKFB3 (Lu et al., 2021). Furthermore, the depletion of PFKFB3 in macrophages abrogates the inhibitory effects of indole on p-p46, p-p65, and inflammatory factors when stimulated by LPS (Ma et al., 2020). However, the involvement of PFKFB3 in macrophages in the pathogenesis of fungal keratitis remains ambiguous, with limited knowledge of its role in this condition; thus, the mechanisms associated with PFKFB3 in fungal keratitis require urgent investigation.

PI3K/AKT is a crucial and classic signaling pathway; the stimulated PI3K leads to the recruitment and activation of the downstream target AKT (Li et al., 2024). Activated AKT, functioning as an upstream target of NF-κB, enhances the phosphorylation of NF-κB and facilitates its translocation into the nucleus, hence boosting the transcription of various inflammation-related genes (Li et al., 2022; Wang Y, et al., 2024). As a key coordinator of immunity and inflammation, NF-κB integrates the functions of the immune and metabolic systems and provokes chronic inflammatory responses (Capece et al., 2022). Researchers have found that PI3K/AKT boosts glucose uptake in macrophages, thereby supplying sufficient substrates for glycolysis (Li et al., 2025). In osteoarthritis, NF-κB activation initiates metabolic alterations linked to cartilage degradation by elevating inflammatory factors, establishing a cross-regulatory network between inflammation and metabolism (Arra et al., 2020). The PI3K/AKT/NF-κB signaling pathway is crucial in regulating glycolysis and inflammation. Our research lays a foundation for exploring the relationship between PI3K/AKT/NF-κB and glycolysis in the pathophysiology of FK.

This paper elucidates the beneficial impacts of PFKFB3 in the pathogenesis of fungal keratitis. Raised PFKFB3 in mice corneas and BMDM after *F. solani* infection prevents the inflammatory cascade and subsequent tissue damage progression to some extent. Furthermore, the regulation of PFKFB3 may involve the participation of the PI3K/AKT/NF-κB p65 signaling pathway. The results of this study are anticipated to facilitate the identification of effective therapeutic targets for fungal keratitis.

## 2 Materials and methods

### 2.1 Preparation of *F. solani*

The standard *F. solani* strain (AS 3.1829) was obtained from the China General Microbiological Culture Collection Center (CGMCC, Beijing, China), inoculated in Sabouraud medium (ThermoFisher Scientific, USA), and incubated at 28 °C for about five days. *Fusarium* spores were harvested utilizing 1 mL of phosphate buffer solution (PBS) (KeyGEN Biotech, Jiangsu, China), and the concentration was diluted to 1 × 10^8^ CFU/mL with PBS following filtration using a sterile filter (100μm, NEST, Wuxi, China). The resultant fungal suspensions were employed as a model for animal infection. In cellular experiments, the spore quantity was adjusted to three times the cell count, and the fungal solution was subjected to heat-inactivation at 95 °C for two hours. This research utilized heat-inactivated spores to replicate *in vitro* infection, as live spore infection can provoke extensive hyphae formation and cell death.

### 2.2 Preparation of animals and FK model

Male BALB/C mice (aged 6 weeks) were purchased from Jinan Pengyue Laboratory Animal Breeding Co., Ltd (Shandong, China). Animal experiments were conducted according to the Association for Research in Vision and Ophthalmology (ARVO). Mice with unblemished, lesion-free corneas were examined using slit-lamp microscopy before model construction. Briefly, mice were anesthetized with 0.6% sodium pentobarbital administered intraperitoneally at a volume of 0.3-0.35 mL, followed by cutting whiskers and eyelashes and applying topical surface anesthetic to the cornea. Following the complete exposure of the mice eyes, the corneal epithelium (about 2.5 mm in diameter) was abraded with a motorized epithelial spatula to guarantee uniformity of the epithelial defect region across all mice. The rat corneal buckle was placed on the ocular surface of the mouse, and 5μL of fungal suspension (1 × 10^8^ CFU/mL) was injected between the buckle and the mouse cornea. The eyelids were secured with a 7–0 suture and removed after 24 hours, and we captured slit lamp pictures on days 1, 3, 5, and 7 post-modeling. Corneas from mice were harvested at designated time points for later experimentation. All experiments followed the guidelines approved by the Animal Ethics Committee of Fujian Medical University (IACUC FJMU 2024-Y-1839).

### 2.3 Corneal intrastromal injection of adeno-associated virus

The AAV-shNC and AAV-shPFKFB3 vectors were designed and produced by Genechem Corporation (Shanghai, China), both utilizing the AAV8 serotype. Mice were systematically sedated via intraperitoneal injection of 0.6% pentobarbital sodium, positioned under a Zeiss operating microscope, and the eyeballs were completely exposed using microforceps. A horizontal incision of approximately 0.5 mm was formed by puncturing the corneal stroma with an 11–0 suture needle in parallel. Next, a 36-gauge needle (WPI, USA) was inserted into the corneal stroma through the horizontal incision. A total of 1.5 μL of AAV-shNC and AAV-shPFKFB3 vectors, each with a viral titer of 1 × 10^9^ vector genomes (vg), were gradually injected into the corneal stroma. Topical ofloxacin eye ointment was applied to prevent infection. Knockdown efficiency was validated using western blot and immunofluorescence analysis 1 month post-injection.

### 2.4 Clinical scoring

The severity of FK was assessed on days 1, 3, 5, and 7 of the infections (*n* = 6/group). The scoring criteria continue to utilize the former scoring system (Wu et al., 2003). The three primary criteria addressed were the dimension and the density of the corneal opaque area, as well as the regularity of its surface. Each criterion was evaluated on a scale from 0 to 4, resulting in a maximum total score of 12. The severity was classified into three categories based on the total score: mild (≤5 points), moderate (6–9 points), and severe (>9 points). To visually evaluate the severity of corneal lesions in accordance with the scoring criteria, three clinicians adopted a double-blind clinical scoring method. The mean score was calculated for analysis after each clinician completed it twice.

### 2.5 Generation of bone marrow-derived macrophage

Six-week-old male BALB/C mice were sacrificed humanely, and the femur and tibia were rinsed with PBS buffer to isolate bone marrow cells, which were then collected by centrifugation at 1000 rpm for 5 minutes. Erythrocytes were subsequently lysed (3 minutes at room temperature) utilizing an erythrocyte lysis solution (Solarbio, Beijing, China). After that, the cells were resuspended in RPMI-1640 medium (KeyGEN Biotech, Jiangsu, China) supplemented with M-CSF (50 ng/mL, Proteintech, Wuhan, China) and transferred to cell culture plates, which were cultured at 37°C with 5% CO_2_. On day 3, the medium was replaced with a fresh medium, including an equivalent concentration of M-CSF. BMDM was effectively induced on day 7 and utilized for further experimental treatments.

### 2.6 BMDM transfection and co-culture with *F. solani*

Induced BMDM were co-cultured with an inactivated fungal suspension (spore-to-cell ratio of 3:1) for 3 hours on day 7, after which the cells were harvested for PCR, western blot, immunofluorescence, and cellular energy metabolism analysis. The cells were transfected on day 7 with Lipofectamine 3000 (Invitrogen, USA) synergized with siNC (200pM, GenePharma, Shanghai, China) and siPFKFB3 (200pM, GenePharma, Shanghai, China) to inhibit the expression of PFKFB3. The cells were co-incubated with the fungal suspension for 3 hours at 24 hours post-transfection, after which RNA was extracted for PCR assay; the cells underwent the same treatment at 48 hours post-transfection and were then analyzed by immunoblotting or immunofluorescence test.

### 2.7 Hematoxylin and eosin staining

Mice eyeballs of different groups (*n* = 3/group) were excised and submerged in 4% paraformaldehyde (Biosharp, Anhui, China). Following dehydration, they were embedded in paraffin and further sectioned into 5μm-thick slices. Next, the sections were stained with H&E and encapsulated with neutral resin. Finally, the slices were examined and photographed using an optical microscope (Leica, Germany).

### 2.8 Quantitative real-time polymerase chain reaction

Corneas at 5 days post-infection and BMDM at 3 hours post-infection (*n* = 3/group) were seized, and mRNA levels of glycolysis-related genes, namely *PFKFB3*, *HK2*, *PFKP*, *Eno2*, *LDHA*, *PGK1*, *Slc2a1*, *Slc16a1*, and *Slc16a3*, along with inflammation-related genes involving *IL-1β*, *IL-6*, *IL-12*, *TNF-α*, *NLRP3*, and *CXCL10* were measured. Per the manufacturer’s instructions, the total RNA was isolated by deploying an isolation kit (TransGen Biotech, Beijing, China). The RNA concentration was quantified based on a microspectrophotometer (Eppendorf, USA), followed by cDNA synthesis by a reverse transcriptase reaction (Vazyme, Nanjing, China). RT-PCR analysis was performed using an SYBR Green mixture (Vazyme, Nanjing, China) on the ABI Prism 7500 (Applied Biosystems, Foster City, CA). Ultimately, data were computed via the comparative cycle threshold approach (2^-ΔΔCt^) and normalized against β-actin. The primers used in this work are presented in **Supplementary Table 1**.

### 2.9 Western blot

Upon harvesting corneas on day 5 post-infection and BMDM on hour 3 post-infection (*n* = 3/group), the samples were homogenized in RIPA lysis solution (Solarbio, Beijing, China), supplemented with phosphatase inhibitors (Beyotime, Shanghai, China) and protease inhibitors (Solarbio, Beijing, China). Electrophoresis separated total protein via 10% or 12.5% SDS-PAGE gels (Epizyme, Shanghai, China) and transferred to PVDF membranes (Millipore, USA). PVDF membranes were blocked with 5% BSA (Solarbio, Beijing, China) for 1 hour at room temperature, followed by overnight incubation with the primary antibody at 4 °C. The next day, the PVDF membranes were washed thrice with TBST and incubated with horseradish peroxidase-conjugated secondary antibody at room temperature for 1 hour. Lastly, the target proteins were quantitatively analyzed using Image J software after being visualized by the ECL reagent (Millipore, USA). The antibodies utilized in this research are presented in **Supplementary Table 2**.

### 2.10 Immunofluorescence staining

7μm frozen sections were prepared after embedding mice eyeballs 5 days post-infection (*n* = 3/group) in OCT gel (Sakura, Tokyo, Japan). In addition, BMDM infected with *F. solani* for 3 hours were collected (*n* = 3/group). Tissue slices or BMDM were fixed in 4% paraformaldehyde and permeabilized with 1% (tissue) or 0.1% (cells) Triton X-100 (Solarbio, Beijing, China). 5% BSA was selected for blocking (at room temperature for 1 hour), succeeded by incubation with the primary antibody (at 4 °C overnight), and then, the fluorescein-conjugated secondary antibody was applied (at room temperature, shielded from light, for 1 hour) the following day. Slides were sealed with Fluoroshield containing DAPI (ab104139, Abcam, UK), and photographs were then processed by ultra-high resolution laser confocal microscopy (ZEISS, LSM880, Germany). The antibodies employed in immunofluorescence are also listed in **Supplementary Table 2**.

### 2.11 Seahorse extracellular flux analysis

Briefly, BMDM were seeded at a density of 8 × 10^4^ cells per well on Seahorse XFp Cell Culture Miniplates (103022-100, Agilent, CA, USA) and incubated overnight at 37 °C in a 5% CO_2_ condition. The next day, the fungal suspension containing 2.4 × 10^5^ spores was applied to infect BMDM for 3 hours. The extracellular acidification rate (ECAR) of BMDM (*n* = 3/group) was detected with the Seahorse XFp HS Mini Metabolic Flux Analyzer (Agilent, CA, USA) in conjunction with the Seahorse XFp Glycolytic Stress Test Kit (103017-100, Agilent, CA, USA). The culture solution in the cell well plates was substituted with XF DMEM medium (103075-100, Agilent, CA, USA) augmented with 2mM glutamine (103579-100, Agilent, CA, USA) in conformity with the manufacturer’s procedure and incubated in a CO_2_-free incubator at 37°C for 45–60 minutes. Besides, additional reagents were added sequentially at the designated concentrations: 10 mM glucose, 1 μM oligomycin, and 50 mM 2-DG. Eventually, ECAR findings were generated by drawing on the Seahorse Wave Desktop software and examined for parameter variations, including non-glycolytic acidification, glycolysis, and glycolytic capacity among distinct groups.

### 2.12 Lactate assay

At 5 days after infection, lactate levels in mouse corneas (*n* = 3/group) were checked using a lactate assay kit (BC2230, Solarbio, Beijing, China) according to the manufacturer’s guidelines. A multifunctional microplate reader (SpectraMax i3x, Molecular Devices, Sunnyvale, USA) gathered the samples’ optical density values at 570 nm, and the lactate content was normalized by sample mass.

### 2.13 Statistical analysis

All experiments in this research were performed a minimum of three times and reported as mean ± standard deviation (SD). The two groups were compared using a two-tailed Student’s t-test through GraphPad Prism software. In comparison, one-way ANOVA was employed for comparisons involving more than two groups. *P* < 0.05 was deemed statistically significant.

## 3 Results

### 3.1 Establishment of FK infection model in mice

**Figure 1:**
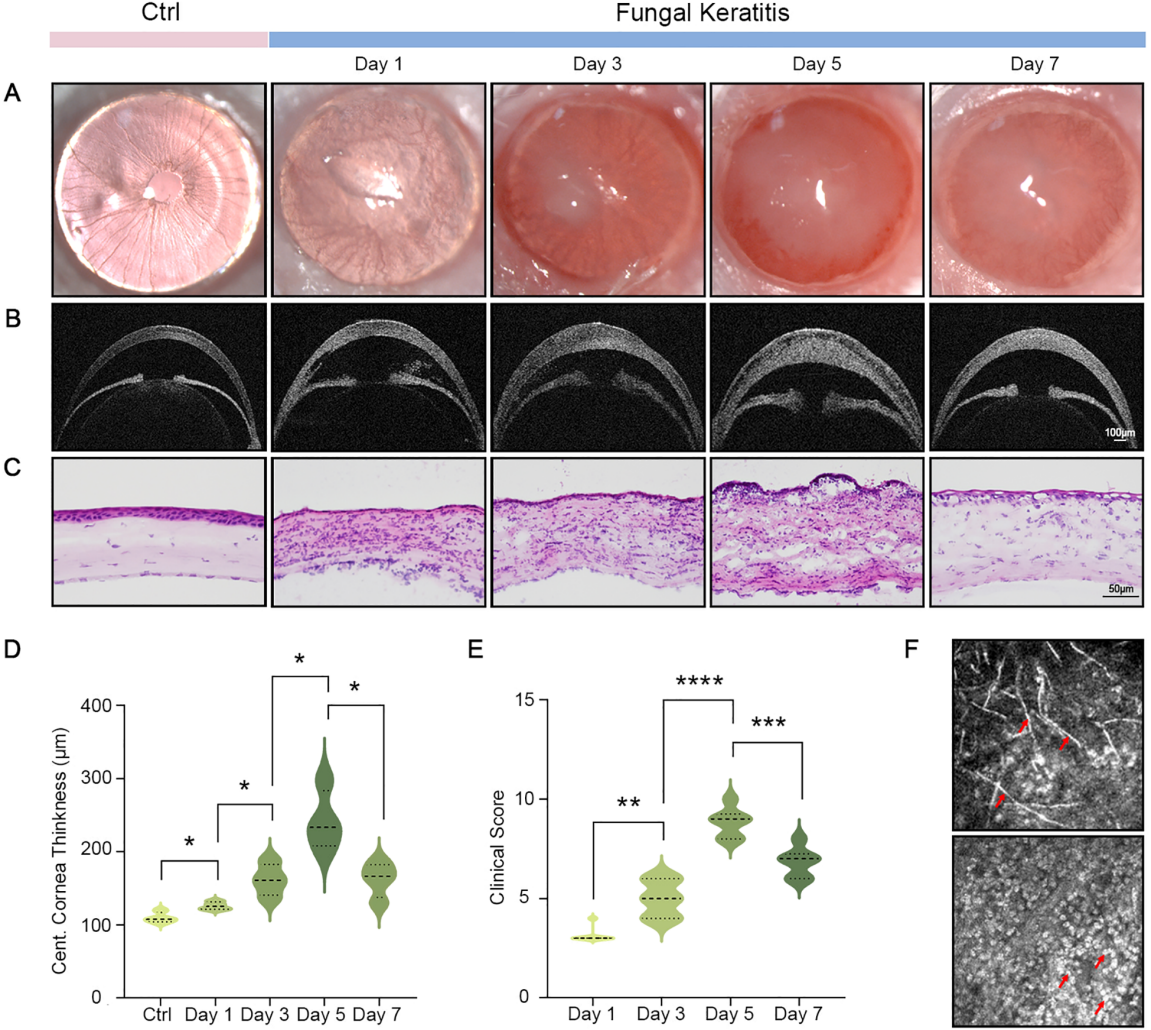
Clinical manifestations of FK associated with *F*. *solani* infection. Slit-lamp images **(A)**, corneal OCT **(B)**, and H&E staining **(C)** in the control group and on days 1, 3, 5, and 7 post *F*. *solani* infection. (Scale bar for corneal OCT: 100 μm; Scale bar for H&E staining: 50 μm) **(D)** Central corneal thickness in normal and FK mice (on days 1, 3, 5, and 7). **(E)** Clinical scores of FK at various time intervals. **(F)** On the 5-day post-infection, confocal microscopy identification of hyphae was highly reflective, and there were a large number of densely distributed inflammatory cells. Red arrows indicate hyphae and inflammatory cells. *P < 0.05, **P < 0.01, ***P < 0.001, ****P < 0.0001.

We developed a fungal keratitis model for mice by infecting *F. solani*. The cornea exhibited edema and slight turbidity on the first day following the fungi infection. Symptoms deteriorated steadily as the disease advanced, with the ulcer’s area expanded. The lesion’s severity peaked on day 5 post-infection, with a gradual reduction of symptoms by day 7 (**Figure 1A**). Corneal OCT revealed anterior chamber exudation on the first-day post-infection, which was progressively reabsorbed. Nonetheless, a notable augmentation in corneal thickness occurred, reaching its zenith on day 5 while diminished by day 7 (**Figures 1B, D**). H&E staining revealed noteworthy infiltration of inflammatory cells at the lesion site, together with corneal tissue looseness, edema, and epithelial defects (**Figure 1C**). The clinical scores of the cornea on days 1, 3, 5, and 7 corresponded with the symptoms of infection (**Figure 1E**). Additionally, a significant quantity of hyphae was found in the cornea, accompanied by notable infiltration of inflammatory cells on day 5 post-infection, as confirmed using confocal microscopy (**Figure 1F**).

### 3.2 Enhanced PFKFB3 signaling triggered during *F. solani* infection

**Figure 2:**
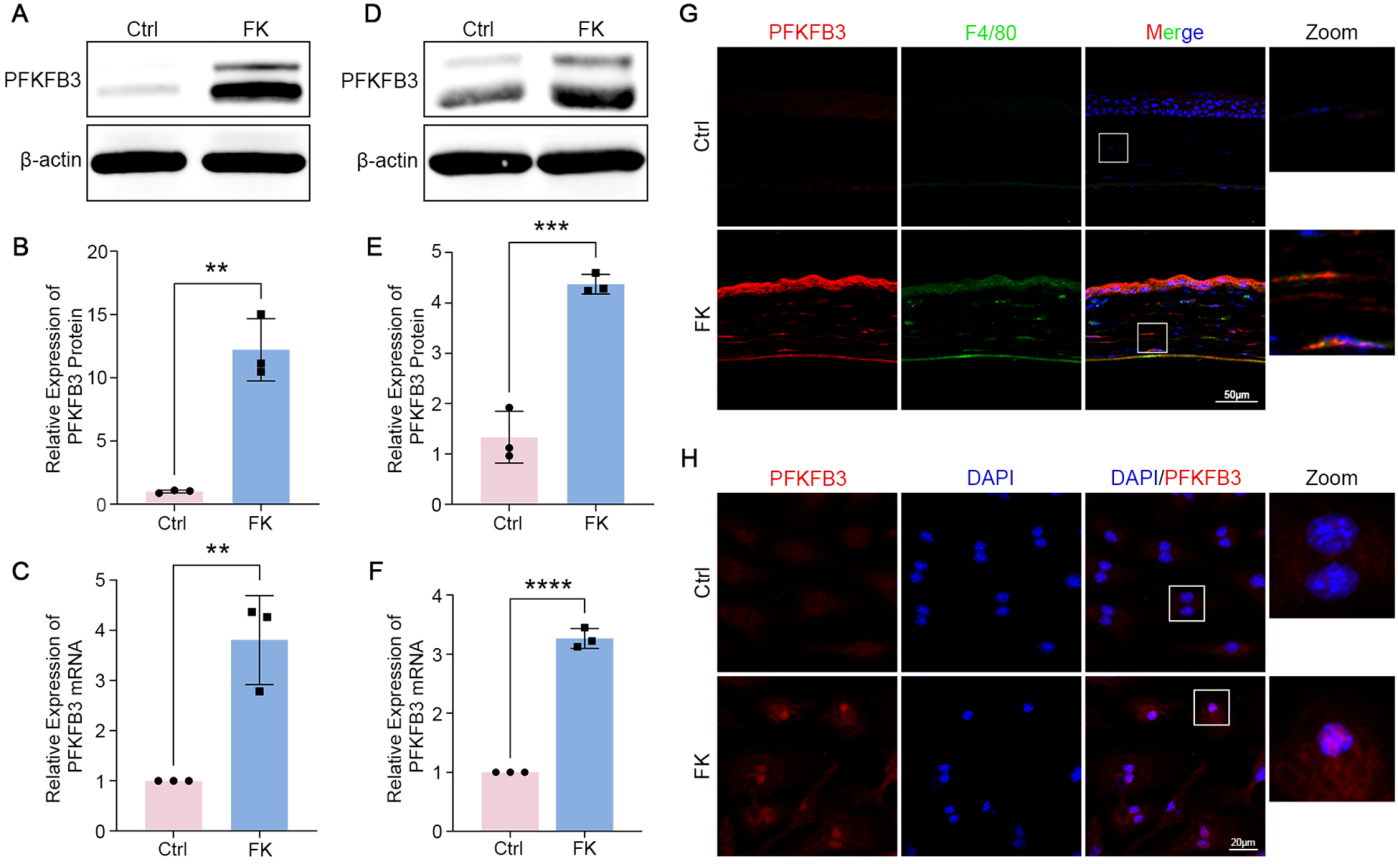
FK correlates with considerably elevated levels of PFKFB3. **(A, B)** PFKFB3 expression in *F*. *solani*-infected corneas was assessed via western blot and quantified using a bar graph. **(C)***PFKFB3* mRNA levels in mice corneas were quantified using RT-PCR. **(D, E)** Western blot analysis was employed to detect PFKFB3 protein levels in BMDM infected with *F*. *solani*. **(F)** RT-PCR test for *PFKFB3* mRNA levels in BMDM. **(G)** Immunofluorescence displaying the expression of PFKFB3 (red) and F4/80 (green) in the corneas of normal and FK mice. Nuclear DNA was labeled with DAPI (blue). Scale bar: 50μm. **(H)** Immunofluorescence research on PFKFB3 (red) in BMDM. Nuclei were stained with DAPI (blue). Scale bar: 20 μm. **P < 0.01, ***P < 0.001, ****P < 0.0001.

To elucidate the pathogenesis of PFKFB3 in FK, we explored its expression in animal and cellular infection models. A noticeable rise in PFKFB3 levels within corneas was noted via western blot, accompanied by a comparative graph of its gray value analysis (**Figures 2A, B**). PCR similarly explained the substantial disparities in *PFKFB3* expression between untreated and infected corneas (**Figure 2C**). To investigate the association between active macrophages in FK and PFKFB3 expression, we conducted double immunofluorescence staining. The findings revealed an impressive accumulation of macrophages in the corneas of fungus-infected mice, with increased PFKFB3 partially co-localizing with F4/80 positive macrophages (**Figure 2G**), reinforcing our hypothesis. Afterward, *in vitro* experiments showed that BMDM co-cultured with *Fusarium* spores displayed analogous alterations, characterized by significantly higher protein levels of PFKFB3 (**Figures 2D, E**). As anticipated, the mRNA transcription of *PFKFB3* was likewise upregulated in BMDM (**Figure 2F**). Immunofluorescence proves that infected BMDM displayed boosted PFKFB3 expression, predominantly localized in the nucleus (**Figure 2H**). The aforementioned data suggest that *F. solani* can proficiently induce PFKFB3 expression in macrophages *in vivo* and *in vitro*.

### 3.3 Potential inflammatory and glycolytic microenvironment in BMDM co-cultured with *F. solani*

**Figure 3:**
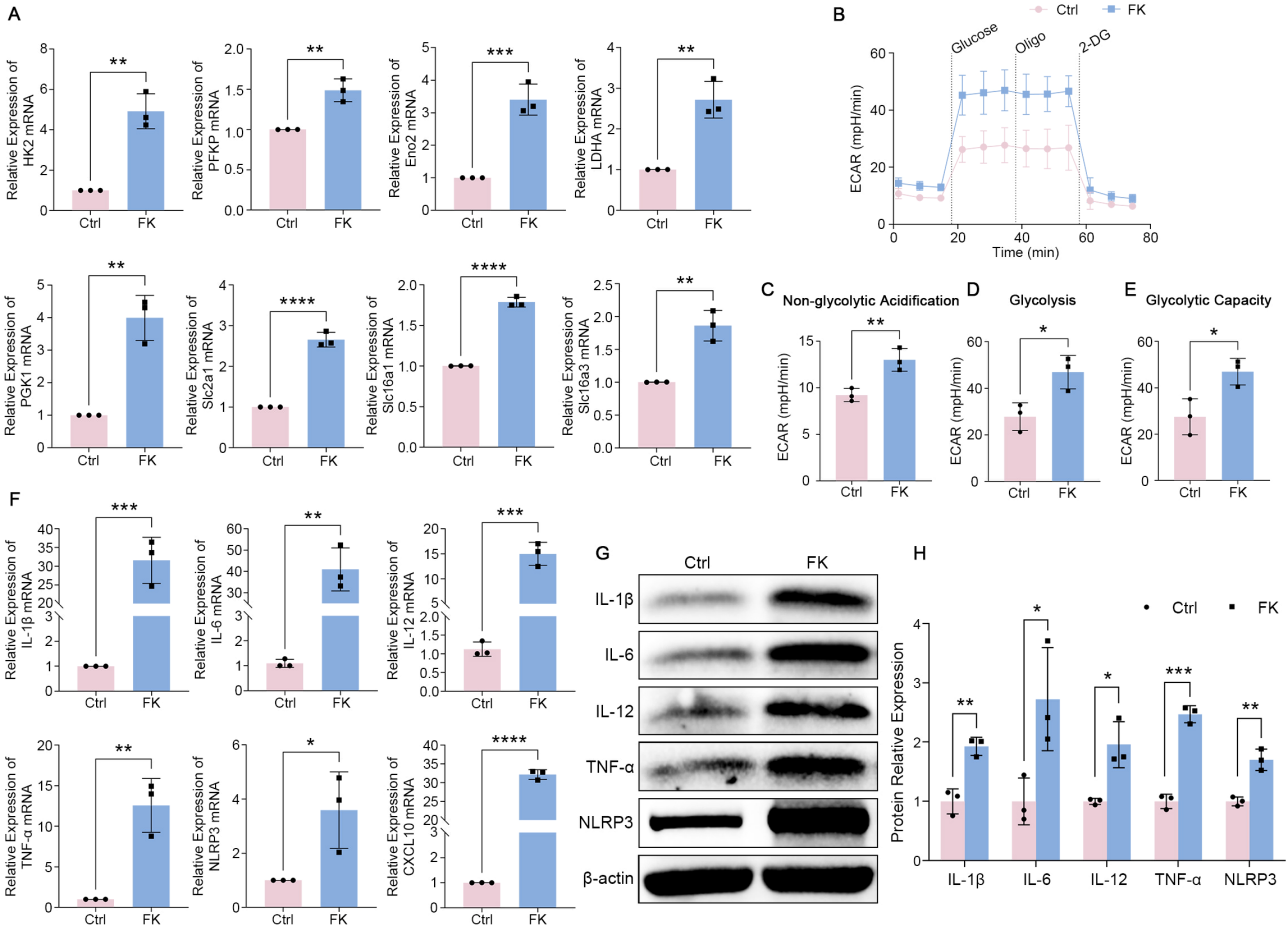
Glycolytic flux and inflammation were stimulated in *F*. *solani*-treated BMDM. **(A)** RT-PCR results of *HK2*, *PFKP*, *Eno2*, *LDHA*, *PGK1*, *Slc2a1*, *Slc16a1*, and *Slc16a3* in BMDM in the presence or absence of *F*. *solani*. **(B)** The ECAR profile in BMDM was evaluated by the Seahorse XFp HS Mini Metabolic Flux Analyzer. As shown, glucose, oligomycin, and 2-DG were administered successively at various time points. **(C–E)** ECAR values were produced through the Seahorse Wave Desktop program to yield findings for non-glycolytic acidification, glycolysis, and glycolytic capacity. **(F)** RT-PCR for checking the expression levels of *IL-1β*, *IL-6*, *IL-12*, *TNF-α*, *NLRP3*, and *CXCL10* across several groups. **(G, H)** Western blot analysis was applied to examine the levels of inflammatory components in BMDM, both with and without infection, followed by gray value analysis. *P < 0.05, **P < 0.01, ***P < 0.001, ****P < 0.0001.

In the meantime, we analyzed the impact of fungal spores on glycolytic flux and inflammation in BMDM. Hence, we exogenously added heat-inactivated spores to BMDM. PCR analysis stated an enormous upregulation of key glycolytic enzymes (such as *HK2*, *PFKP*, *Eno2*, *LDHA*, and *PGK1*), along with glucose/lactic acid transporter-associated genes (*Slc2a1*, *Slc16a1*, and *Slc16a3*) in BMDM (**Figure 3A**). Then, we sequentially administered glucose, oligomycin, and 2-DG to *Fusarium*-treated BMDM and assessed their ECAR levels. The outcomes pointed out that the treated group showed a higher profile of ECAR, with significant amplification of non-glycolytic acidification, glycolysis, and glycolytic capacity measures (**Figures 3B–E**). Later on, we observed modifications in the inflammatory microenvironment, and PCR suggested a greater quantity of inflammatory factors (*IL-1β*, *IL-6*, *IL-12*, *TNF-α*, *NLRP3*, and *CXCL10*) was generated in the infected group versus the control group (**Figure 3F**). In addition, the protein concentrations of pro-inflammatory components were markedly elevated in infected BMDM (**Figures 3G, H**). In conclusion, *F. solani*-stimulated BMDM exhibited elevated glycolytic activity and inflammatory responses.

### 3.4 Enhanced glycolytic flux and inflammation in murine FK

**Figure 4:**
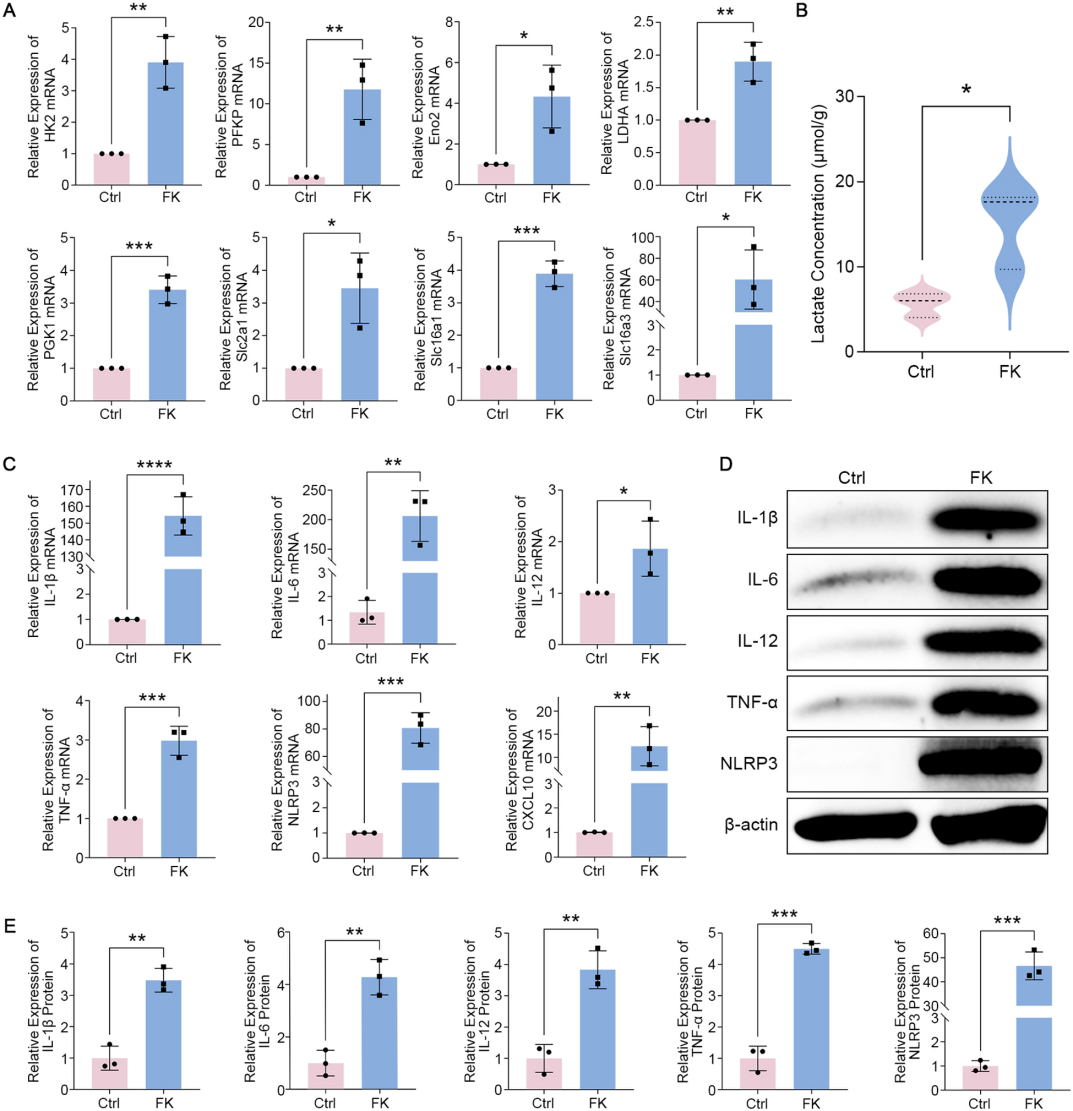
Glycolysis and inflammation are abnormally provoked in FK mice. **(A)** RT-PCR investigation of mRNA transcript levels for *HK2*, *PFKP*, *Eno2*, *LDHA*, *PGK1*, *Slc2a1*, *Slc16a1*, and *Slc16a3* in corneas. **(B)** Lactate concentration in infected and uninfected corneas was measured using a Lactate Assay. **(C)** mRNA expression levels of *IL-1β*, *IL-6*, *IL-12*, *TNF-α*, *NLRP3*, and *CXCL10* among various groups. **(D, E)** Western blot examination of IL-1β, IL-6, IL-12, TNF-α, and NLRP3 proteins in corneas. *P < 0.05, **P < 0.01, ***P < 0.001, ****P < 0.0001.

Corneas from fungi-infected mice were collected to corroborate our results in the cellular model. PCR verified that the glycolytic enzymes and glucose/lactate transporter-related genes depicted in **Figure 3** showcased comparably substantial rises (**Figure 4A**). Lactate levels in FK were markedly elevated compared to those in the normal group (**Figure 4B**). We also assessed the degree of inflammation in the infected group. Unsurprisingly, the inflammatory mediators’ mRNA and protein levels depicted in **Figure 3** were profoundly up-regulated in FK (**Figures 4C–E**). In brief, glycolysis and inflammatory levels were comparably triggered in the mice FK model.

### 3.5 PFKFB3 depletion enhances the inflammatory cascade in BMDM

**Figure 5:**
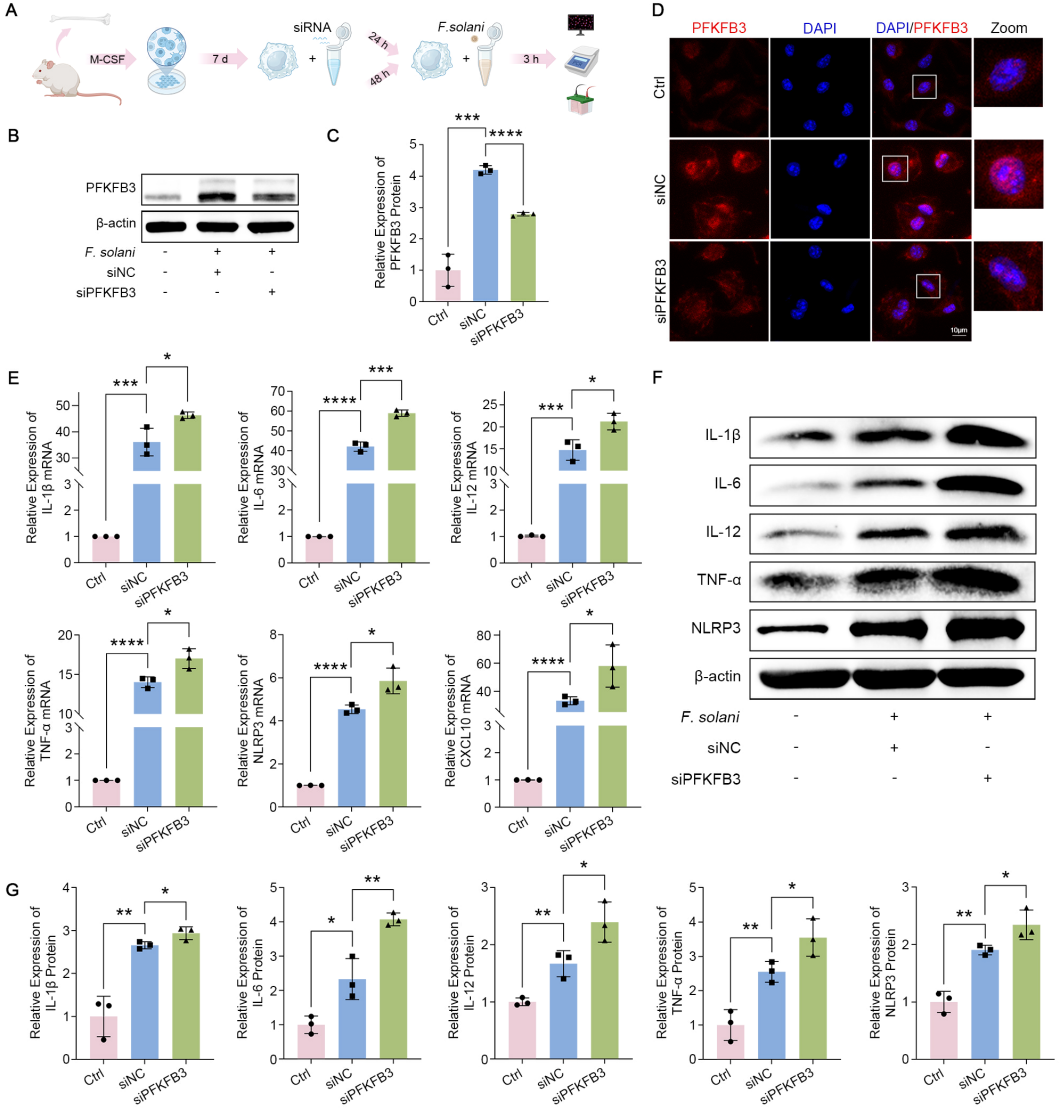
Inhibition of PFKFB3 intensifies the inflammatory cascade in *F. solani*-treated BMDM. **(A)** Schematic illustration of the BMDM culture, siRNA transfection, and co-cultivation with *F. solani*. The knockdown efficacy of PFKFB3 in BMDM was assessed by western blot **(B, C)** and immunofluorescence **(D)**. Scale bar: 10 μm. **(E)** Transcript levels of inflammatory factors (including *IL-1β*, *IL-6*, *IL-12*, *TNF-α*, *NLRP3*, and *CXCL10*) in BMDM across multiple pretreatment groups during fungal infection were detected via RT-PCR. **(F, G)** Western blot was performed to identify the protein levels of IL-1β, IL-6, IL-12, TNF-α, and NLRP3 in BMDM, with quantification achieved using Image J. *P < 0.05, **P < 0.01, ***P < 0.001, ****P < 0.0001.

To figure out the exact regulatory function of PFKFB3 in fungal infections, we intervened in its expression. We suppressed the expression of PFKFB3 in BMDM harnessing siRNA (**Figure 5A**). The silencing efficacy of siPFKFB3 was evaluated by western blot and immunofluorescence, confirming a substantial reduction of PFKFB3 (**Figures 5B–D**). Next, we focused on the impact of PFKFB3 depletion on subsequent inflammatory responses. Our observations showed that the amounts of inflammatory mediators (including IL-1β, IL-6, IL-12, et al) were markedly increased in the siNC group relative to the normal group, matching the prior data in **Figure 3**. Unexpectedly, the expression of mRNAs for inflammatory factors was found to be considerably raised obeying the knockdown of PFKFB3 (**Figure 5E**). The western blot results reinforced this outcome (**Figures 5F, G**). These effects underscore the protective role of PFKFB3 activation in FK pathogenesis, with its upregulation mitigating further intensifying the inflammatory cascade response.

### 3.6 Blockade of PFKFB3 exacerbates inflammation and lesions in FK

**Figure 6:**
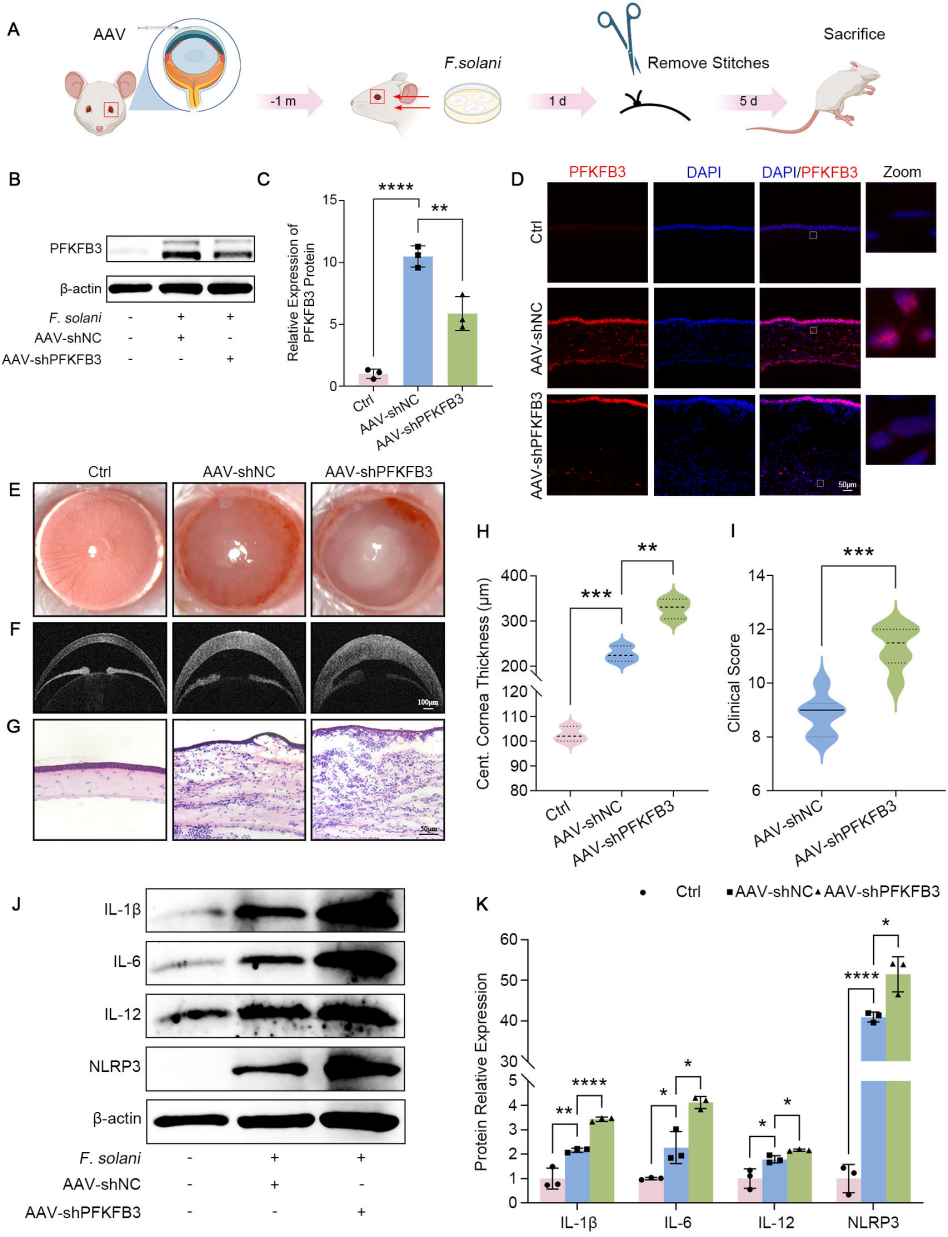
Targeting PFKFB3 with AAV exacerbated the severity of FK. **(A)** Schematic timeline of AAV and *F. solani* infection in the FK murine model. PFKFB3 was effectively silenced in the cornea, as verified by western blot **(B, C)** and immunofluorescence **(D)**. Scale bar: 50 μm. Slit-lamp images **(E)**, corneal OCT pictures **(F)**, and H&E staining **(G)** results from several groups. (Scale bar for corneal OCT: 100 μm; Scale bar for H&E staining: 50 μm). **(H)** Measurement of central corneal thickness in mice. **(I)** Clinical scores of AAV-shNC and AAV-shPFKFB3 pretreated FK were evaluated. **(J, K)** Western blot detection of IL-1β, IL-6, IL-12, and NLRP3 protein levels in mice cornea in various treatment groups was quantified using Image J. *P < 0.05, **P < 0.01, ***P < 0.001, ****P < 0.0001.

We explored the influence of suppressing PFKFB3 on FK through corneal intrastromal injection of AAV-shPFKFB3. The FK model was built 1 month after AAV injection, and the cornea was collected on day 5 post-infection (**Figure 6A**). PFKFB3 was successfully knocked down, as confirmed by western blot and immunofluorescence (**Figures 6B–D**). Following this, we evaluated the severity of the corneal infection. As anticipated, following the blockade of PFKFB3, the cornea exhibited exacerbated lesions, characterized by an enlarged ulcerative region, heightened corneal opacity (**Figure 6E**), aggravated corneal edema (**Figures 6F, H**), and intensified infiltration of inflammatory cells (**Figure 6G**). Simultaneously, the clinical scores in the AAV-shPFKFB3 group exceeded those in the AAV-shNC group (**Figure 6I**). Moreover, the protein levels of pro-inflammatory cytokines (IL-1β, IL-6, IL-12, and NLRP3) were also highly expressed in the AAV-shPFKFB3 group (**Figures 6J, K**). Collectively, these investigations demonstrate that PFKFB3 knockdown similarly intensified the FK inflammatory response and worsened damage to the cornea.

### 3.7 PFKFB3 deficiency boosting the PI3K/AKT/NF-κB p65 signaling pathway

**Figure 7:**
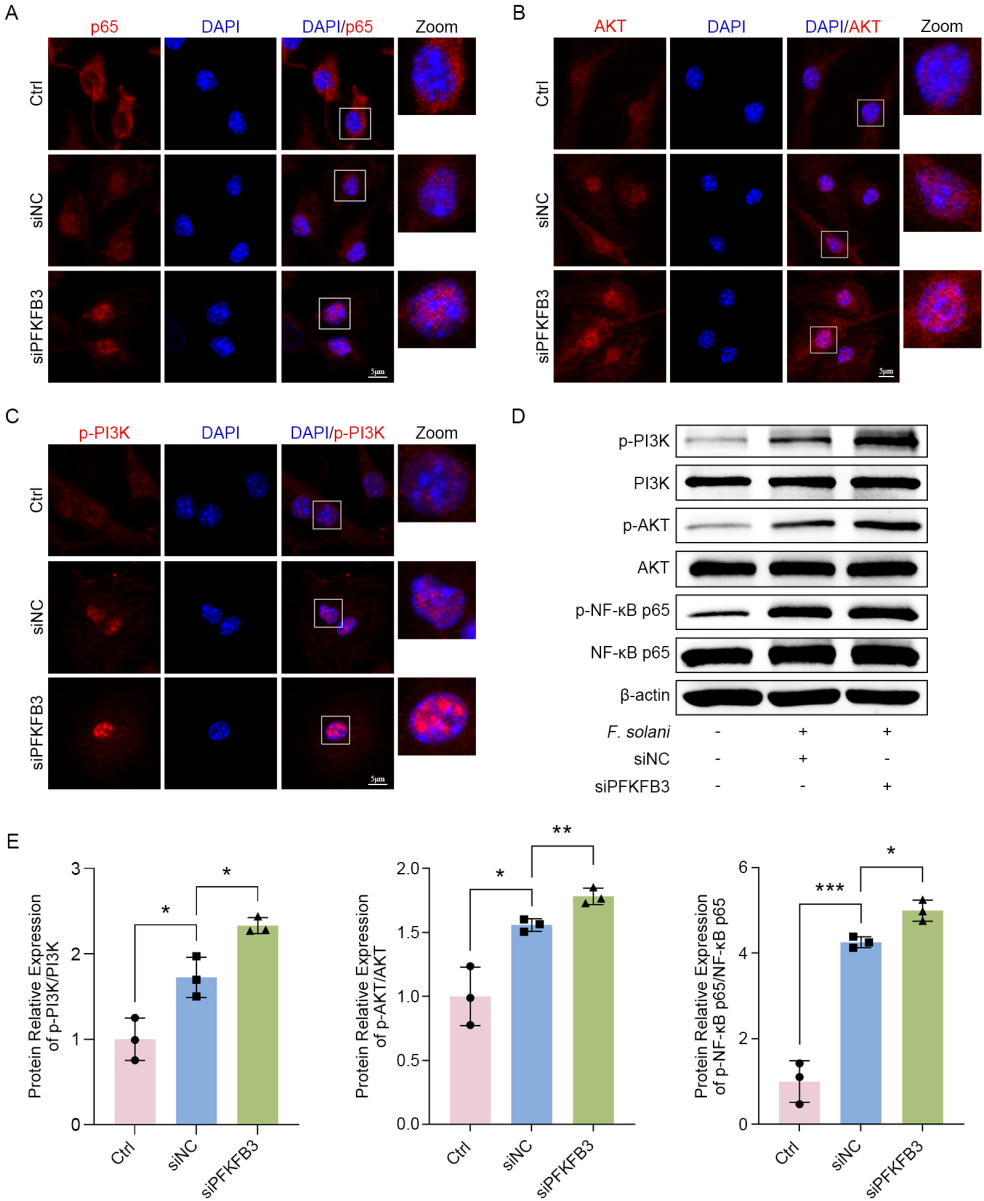
Fungal infection accompanied by PFKFB3 deficiency further triggers the PI3K/AKT/NF-κB p65 signaling pathway. **(A–C)** Immunofluorescence was employed to locate and quantify p65 (red), AKT (red), and p-PI3K (red) in BMDM under different conditions, with nuclear staining executed using DAPI (blue). Scale bar: 5 μm. **(D, E)** Western blot analysis was conducted to assess the phosphorylation levels of PI3K, AKT, and NF-κB p65 in different groups of BMDM. *P < 0.05, **P < 0.01, ***P < 0.001.

However, the mechanism by which PFKFB3 modulates inflammation in FK is yet to be understood. To address this issue, we inspected the signaling pathways potentially implicated in the depletion of PFKFB3. We targeted the PI3K/AKT/NF-κB p65 signaling pathway, which was linked to FK inflammation in our prior research (Huang et al., 2022; Tang et al., 2022). The immunofluorescence results claimed a heightened nuclear translocation of PI3K/AKT/NF-κB p65 in the siNC group relative to the untreated group. Nevertheless, the nuclear translocation of these targets was notably enhanced following PFKFB3 deficiencies (**Figures 7A–C**). On top of that, we launched western blot to check out the expression of phosphorylated proteins. Western blot backed up immunofluorescence discoveries, pointing to that fungi-infection induced vital phosphorylation level of NF-κB p65, AKT, and PI3K in BMDM, these targets were notably enhanced following PFKFB3 deficiencies (**Figures 7D, E**). Taken together, these results underscore that PFKFB3 may rely on the PI3K/AKT/NF-κB p65 signaling pathway to modulate inflammation.

## 4 Discussion

Fungal keratitis constitutes an immense public health and safety issue, resulting in corneal blindness. A primary causal component of fungal keratitis is phyto-trauma to the cornea, while inappropriate wearing of corneal contact lenses and excessive application of steroid drugs are also noteworthy culprits (Yu et al., 2024). Fusarium spores infiltrate the corneal epithelium, germinate within the stroma, and develop mycelium, triggering an inflammatory reaction in the surrounding tissues and causing severe corneal infections (Abbondante et al., 2023). The eventual outcome of FK is contingent upon the equilibrium between the local inflammatory immunological response to the trauma and the organ’s reparative processes, with a suitable immune response essential for eradicating the fungus. Macrophages can regulate and restrict fungal infections by phagocytosis and killing of fungus, in addition to collaborating with T cells (Casadevall, 2022). Our research findings revealed a notable up-regulation of F4/80 staining positivity in the infected cornea, indicating that FK may augment the immune response via macrophage activation, aiding in the resistance against tissue damage caused by fungal spores. Yet, the inflammatory immune response amplified by macrophages worsens cornea injury and promotes ulceration. The local inflammatory immune response is deemed dysregulated, resulting in continuous inflammatory cell infiltration and destroying the corneal structure.

Glycolysis, a crucial metabolic route, is a biological process that harnesses glucose for energy generation under anaerobic circumstances, forming the metabolites pyruvate and lactate (Zhou et al., 2022; Cheng et al., 2021). Metabolic reprogramming is an organism’s adaptation to its milieu and occurs in multiple immune cells containing macrophages, T cells, and dendritic cells (Li et al., 2023). In parallel, glycolysis facilitates cancer proliferation, immune evasion, angiogenesis, and other pathological alterations (Feng et al., 2020). Numerous rate-limiting enzymes and glucose transporter proteins involved in glycolysis fundamentally contribute to glucose absorption, the regulation of glycolytic flux, and energy consumption (Veys et al., 2020). Hexokinase 2 (HK2) modulates mitochondrial metabolism, moderating inflammatory responses and cell death, thus safeguarding mice from DSS-induced colitis (Hinrichsen et al., 2021). Some scholars have found elevated mRNA abundances of enolase 2 (γ-enolase, Eno2) are correlated with the onset of neutrophilic inflammation and contribute to the pathophysiology of impaired lung function in severe asthma (Winter et al., 2021). Phosphoglycerate kinase 1 (PGK1) enhances M1 polarization and inflammatory responses in microglia generated by ischemia/reperfusion injury through the modulation of glycolysis and the synthesis of p300 modulated by H3K27 acetylation (Cao et al., 2023). Prior research has established the essential role of glycolysis in inflammation progression; nevertheless, the potential relevance of heightened glycolytic flux in the pathogenesis of fungal keratitis remains unexplored. Our data confirmed an alarming rise in glycolysis levels in corneas and BMDM infected with *Fusarium*, characterized by advanced mRNA levels of glycolytic enzymes such as *HK2*, *PGK1*, *Eno2*, et al, along with a significant upregulation of genes encoding glucose/lactic acid transporter proteins (*Slc2a1*, *Slc16a1*, and *Slc16a3*). Besides, lactate concentrations were radically high in ailing corneas. Lactic acid, a byproduct of glycolysis, is a category of detrimental chemicals (Wang et al., 2019). Simultaneously, we accomplished ECAR assays on BMDM utilizing cellular energy metabolism instrumentation, and observations show that infected BMDM displayed surprisingly heightened ECAR parameters (including non-glycolytic acidification, glycolysis, and glycolytic capacity). We found that infected macrophages with *F. solani* have raised glycolysis, implying that activated immune cells may depend on glycolysis to sustain energy requirements during fungal keratitis.

Mounting evidence proposes that PFKFB3, an indispensable promoter of glycolysis, propelled glycolysis and heightened inflammatory responses in macrophages (Chen et al., 2024). The depletion of PFKFB3 in endothelial cells causes impaired macrophage M2 polarization, thereby impacting muscle revascularization and regeneration (Zhang et al., 2020). In PFKFB3-deficient myeloid cells, glycolytic metabolite levels are significantly reduced, which restricts the transition from macrophages to myofibroblasts, ultimately diminishing renal fibrosis (Yang et al., 2023). Actually, PFKFB3 is widely expressed in human tissues and is known to be overexpressed in numerous inflammatory disorders. In recent years, PFKFB3 has emerged as a compelling therapeutic target due to its role as a vital rate-limiting enzyme in glycolysis, prompting us to designate it as the focal point of this investigation. Our observations reveal that the level of PFKFB3 is exceptionally high in the *Fusarium* action microenvironment, both *in vivo* and *in vitro*. The co-localization of PFKFB3 with macrophages in the mice cornea further substantiates the overall augmentation of PFKFB3 in activated macrophages, partially explaining the enhanced glycolysis in FK. Based on this result, we postulate that macrophage glycolysis plays a key part in the mechanism of FK.

In acute lung injury, aggravated histopathology and pushed production of pro-inflammatory cytokines IL-6 and CXCL1 following treatment with the PFKFB3 inhibitor 3PO, illustrating that PFKFB3 in the alveolar epithelium serves a role in maintaining alveolar integrity by augmenting glycolytic (Vohwinkel et al., 2022). In addition, scholars have discovered that a high-fat diet diminished PFKFB3 expression in the intestinal epithelium, consequently amplifying inflammation; conversely, a high-glucose diet mitigated LPS-induced intestinal inflammation by enhancing PFKFB3 expression (Botchlett et al., 2016). Zhu et al. reported that blocking PFKFB3 enhances the synthesis of inflammatory mediators (such as TNF-α and IL-1β), deteriorating local and systemic insulin resistance (Zhu et al., 2021). The findings above align with the conclusions of our paper, revealing that the silencing of BMDM and murine corneal PFKFB3 expression using siRNA and AAV intensified tissue inflammation provoked by *F. solani*. Blockade of PFKFB3 enhanced the production of inflammatory mediators, including IL-1β, IL-6, IL-12, and NLRP3, precipitated a more grave inflammatory cascade, and resulted in worsening corneal injuries characterized by intensified localized ulceration, substantial corneal edema thickening, and heightened inflammatory cell infiltration. According to our findings, PFKFB3 inhibition sufficiently exacerbates FK inflammation, proving that PFKFB3 is vital for delaying *F. solani*-induced inflammation. These data find that PFKFB3-mediated glycolytic flux and metabolic condition in macrophages may directly affect inflammation and cellular function, hence serving as a shield in FK pathogenesis. Interestingly, a study on hypoxia-induced upregulation of PFKFB3 reveals that suppressing the upstream target HIF-1α or administering the pharmacological inhibitor PFK158 to decrease PFKFB3 expression can postpone the advancement of atherosclerosis (Wang X, et al., 2024). Similarly, pharmacological suppression of PFKFB3 elicits anti-inflammatory and antifibrotic effects in experimental pulmonary fibrosis, concurrently diminishing NLRP3 activation (Liu C, et al., 2024). These findings contradict our results, indicating that PFKFB3 functions within a multifaceted regulatory network. Conversely, research has validated that administration of the PFKFB3 agonist meclizine facilitates endometrial repair in mice, with PFKFB3 exhibiting an inverse connection with the extent of endometrial fibrosis (Mao et al., 2024). This may stem from variations in injury-specific and cell types, necessitating further inquiry into the underlying mechanisms. In summary, our data indicate that proper activation of PFKFB3 significantly decelerates the advancement of *F. solani* keratitis. However, future research needs to examine the pivotal role of PFKFB3 overexpression in inflammation and keratitis consequences. In the context of *F. solani* infection, the impact of increased PFKFB3 on inflammation and corneal ulceration, as well as the underlying mechanisms, necessitates additional exploration. Overall, our study signifies that PFKFB3 may be a promising target for treating fungal keratitis. Clinically, can we infer if variations in PFKFB3 expression are present across patients with differing severities of *F. solani* keratitis? If such differences exist, are they restricted exclusively to the local corneal tissue? Could this be a novel drug target for antifungal? Therefore, extra research is required to clarify the feasibility of therapeutically modifying PFKFB3 in FK.

The PI3K/AKT signaling pathway involves essential physiological activities containing apoptosis, metabolism, angiogenesis, and the cell cycle, facilitating glucose uptake and utilization while limiting lipid synthesis to sustain an ordinary metabolic state (Tian et al., 2023; Xiao et al., 2022). In hepatocellular carcinoma, PI3K/AKT accelerates glucose absorption in cancer cells by stimulating the synthesis of GLUT1 and GLUT4 (Feng et al., 2020). It has been previously proven that PI3K/AKT benefits glycolysis and lactate production by upregulating PFKFB3 levels (Hu et al., 2020). The overexpression of PFKFB3 in osteoarthritis promotes the survival of cartilage tissues and chondrocytes via the PI3K/AKT/CHOP signaling pathway, thereby conferring protective effects on cartilage (Qu et al., 2016). The induction of the nuclear factor κB (NF-κB) pathway benefits the transcription of lots of inflammation-associated genes, like TNF-α, IL-6, and IL-1β (Wang Y, et al., 2024). In addition, PFKFB3 controls inflammation by accelerating NF-κB activation in inflammatory circumstances such as acute lung injury (Wang et al., 2019). In PFKFB3-deficient macrophages, the inhibitory impact of indole on LPS-induced p-p46 and p-NF-κB p65 was nullified, lowering the efficacy of indole in the treatment of NAFLD (Ma et al., 2020). The disruption of PFKFB3 in macrophages could intensify diet-induced inflammation in white adipose tissue and insulin resistance via augmenting NF-κB p65 phosphorylation (Zhu et al., 2021). Our recent research indicated that *F. solani* keratitis activates the PI3K/AKT/NF-κB p65 signaling pathway, and pharmacological inhibition of PI3K/AKT with LY294002 effectively reduces the transcriptional levels of IL-1β, IL-6, and TNF-α (Huang et al., 2022; Tang et al., 2022). Han et al. revealed that blocking the NF-κB also mitigates corneal damage and inflammatory responses in FK (Han et al., 2023). In light of the aforementioned, we hypothesized that the inflammation intensified by PFKFB3 reduction in FK is mediated by the PI3K/AKT/NF-κB p65 signaling pathway. The data indicated that the knockdown of PFKFB3 expanded *Fusarium*-induced PI3K, AKT, and NF-κB p65 activation while simultaneously promoting their nuclear translocation. Our results speculated that the PI3K/AKT/NF-κB p65 signaling pathway contributes to the protective function of PFKFB3 on fungal keratitis. The TLR pathway, functioning as an upstream regulator of NF-κB transcription, accelerates the macrophage response to fungal infection upon activation (Kawai and Akira, 2007; Pamer, 2008). Previous studies have shown that TLR4 activation effectively facilitates the production of proinflammatory cytokines in fungal keratitis, making TLR4 a key target for modulating the inflammatory response in this condition (Huan et al., 2020; Luan et al., 2023; Xu et al., 2018). Nonetheless, it remains uncertain whether fungal antigens modulate PFKFB3 through TLR-mediated mechanisms. Therefore, future studies are required to explore the upstream targets of *F. solani*’s impact on PFKFB3, investigating whether it involves the TLR pathway or other intricate regulatory networks.

However, our study has some limitations. First, in animal experiments, AAV-shPFKFB3 is not a cell-specific AAV vector, indicating the potential for concurrent inhibition of several cell populations. Future studies are required to elaborate which cell populations demonstrate greater suppression by AAV-shPFKFB3 in *F. solani* keratitis, such as by detecting co-localization of the target gene with cell-specific markers or sorting distinct cell populations to assess knockdown efficacy. Additionally, detailed investigations are required to explore the correlation between the exacerbated corneal lesions following PFKFB3 deficiency and fungal burden, along with the underlying mechanisms. Moreover, while prior research has identified correlations between the PI3K/AKT/NF-κB pathway and antifungal inflammatory immune responses, the crucial role of PFKFB3 in FK inflammation through PI3K/AKT/NF-κB-dependent mechanisms, particularly considering various upstream targets and infections by different fungal strains, necessitates further exploration.

This study reveals the involvement of PFKFB3 in modulating corneal inflammation following fungi infection. More crucially, we defined the connection between PFKFB3 and the PI3K/AKT/NF-κB p65 signaling pathway and predicted that PFKFB3 regulates pro-inflammation in a PI3K/AKT/NF-κB p65-dependent manner, underlining a significant association among glucose metabolism and inflammation. Future research should address whether PFKFB3’s modulation of inflammation through the PI3K/AKT/NF-κB p65 pathway is contingent upon shifts in overall glycolytic flux.

## 5 Conclusion

**Figure 8:**
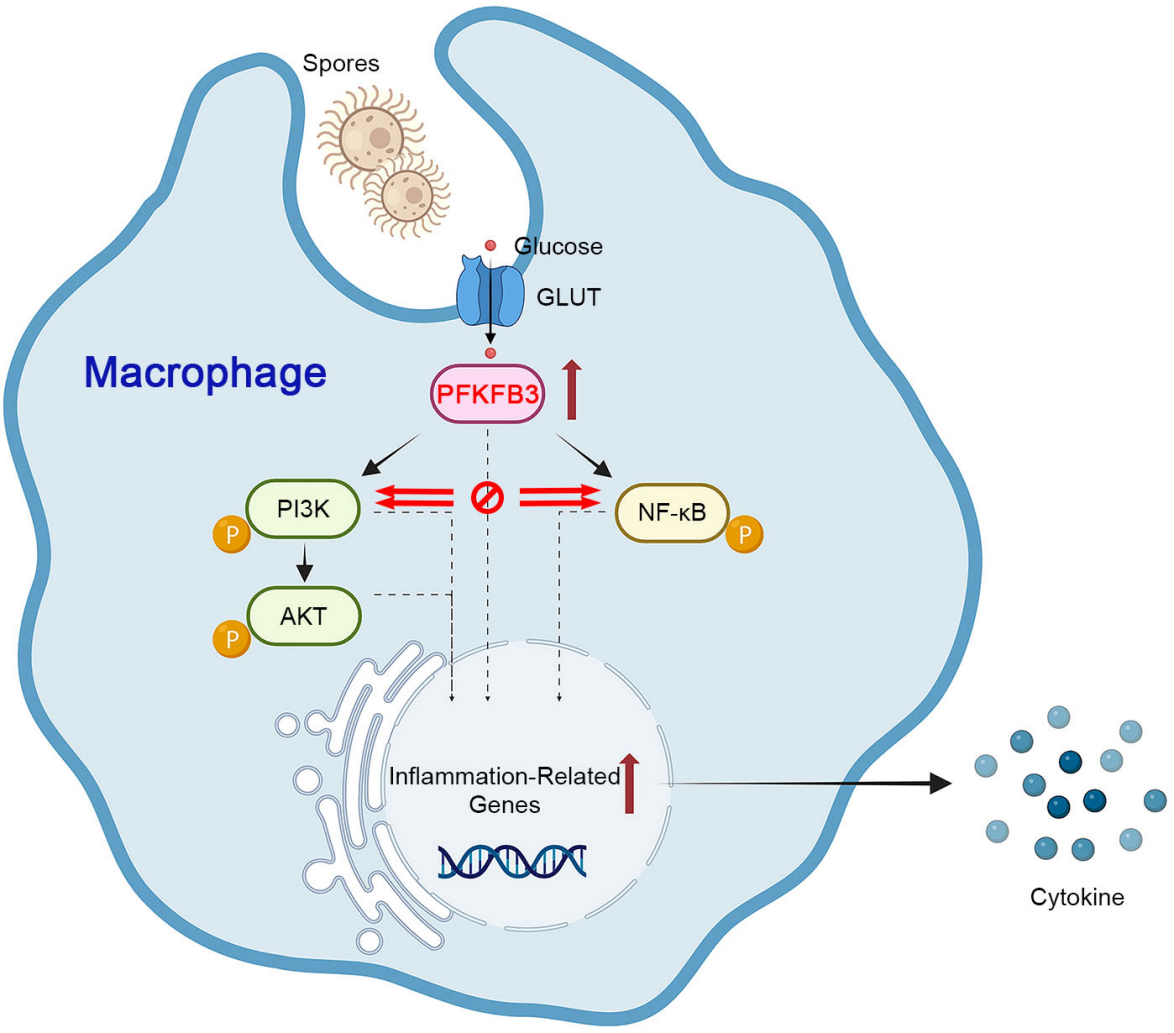
Schematic representation of PFKFB3 function in *F. solani*-infected macrophages.

In summary, we verified boosted glycolytic flux in fungal keratitis. Impairing the critical glycolytic enzyme PFKFB3 could exacerbate inflammation in FK via further activating the PI3K/AKT/NF-κB p65 pathway (**Figure 8**). The activation of PFKFB3 by *F. solani* seems to constitute a defensive mechanism against excessive inflammation. We conclude that targeting macrophage PFKFB3 in fungal keratitis may be a unique therapeutic approach for addressing this illness.

## Data Availability

The original contributions presented in the study are included in the article/**Supplementary Material**. Further inquiries can be directed to the corresponding author.
