# Supplementary material for: PFKFB3 alleviates the advancement of Fusarium solani keratitis by attenuating macrophage inflammation
Source: Front Cell Infect Microbiol. 2025 Dec 4;15:1623027. doi: 10.3389/fcimb.2025.1623027 (PMC12711700; doi:10.3389/fcimb.2025.1623027)
Supplement: Supplementary file 1 [file Table1.docx]

Supplementary Table 1. Primers sequences used in this work

| Gene | Forward | Reverse |
| --- | --- | --- |
| *PFKFB3* | AACGCCTGGAGCCTGTGAT | GGACAGTGTGGAGCGGACAT |
| *HK2* | CTCAGAGCGCCTCAAGACAAG | GCACACCTCCTTCACAATGATG |
| *PFKP* | GTGGCACAGACATGACCATTG | CGCTCACCAAGGCCAAGTAA |
| *Eno2* | CTGCAACTGTTTGCTGCTCAA | ACGACAAGATCAGCAATGAACGT |
| *LDHA* | GTTCATCATTCCCAACATTGTCA | GCTGAATCCAGATTGCAACCA |
| *PGK1* | CTCCATGGTGGGTGTGAATCT | ATCAGCTGGATCTTGTCTGCAA |
| *Slc2a1* | CACTGCACCAGCTGGGAAT | GGCAGAAGGGCAACAGGATA |
| *Slc16a1* | GGCAGCCGTCCAGTAATGAT | AGTCAGAGCTGGGTTCAAGTTGA |
| *Slc16a3* | TCTTTGCGTCCCTGGGAAT | GGCGACGCTTGTTGAAGTATC |
| *IL-1β* | CTTTCCCGTGGACCTTCCA | CTCGGAGCCTGTAGTGCAGTT |
| *IL-6* | ACCACTCCCAACAGACCTGTCT | CAGATTGTTTTCTGCAAGTGCAT |
| *IL-12* | TCACAACCATCAGCAGATCATTC | GGATGCAGAGCTTCATTTTCACT |
| *TNF-α* | ACAAGGCTGCCCCGACTAC | TGGGCTCATACCAGGGTTTG |
| *NLRP3* | CTGCGGACTGTCCCATCAAT | AGGTTGCAGAGCAGGTGCTT |
| *CXCL10* | GGTCCGCTGCAACTGCAT | GGATTCAGACATCTCTGCTCATCA |
| *β-actin* | ACGGCCAGGTCATCACTATTG | AGAGGTCTTTACGGATGTCAACGT |
