# Supplementary material for: PFKFB3 alleviates the advancement of Fusarium solani keratitis by attenuating macrophage inflammation
Source: Front Cell Infect Microbiol. 2025 Dec 4;15:1623027. doi: 10.3389/fcimb.2025.1623027 (PMC12711700; doi:10.3389/fcimb.2025.1623027)
Supplement: Supplementary file 2 [file Table2.docx]

Supplementary Table 2. Antibodies used in this study

| Antibodies | Source | Identifier | Application and Dilution |
| --- | --- | --- | --- |
| anti-PFKFB3 | Abcam | ab181861 | WB, 1:1000 |
| anti-PFKFB3 | Proteintech | CL594-13763 | IF, 1:200 |
| anti-IL-1β | Cell Signaling Technology | 31202S | WB, 1:1000 |
| anti-IL-6 | Abcam | ab229381 | WB, 1:1000 |
| anti-IL-12 | Abcam | ab131039 | WB, 1:1000 |
| anti-TNF-α | Proteintech | 60291-1-Ig | WB, 1:1000 |
| anti-NLRP3 | Abcam | 270449 | WB, 1:1000 |
| anti-phospho-PI3K | Affinity | AF3242 | IF, 1:200 |
| anti-phospho-PI3K | Abcam | 191606 | WB, 1:500 |
| anti-PI3K | Abcam | 182651 | WB, 1:1000 |
| anti-phospho-AKT | Cell Signaling Technology | 4060S | WB, 1:2000 |
| anti-AKT | Cell Signaling Technology | 9272S | WB, 1:1000; IF, 1:200 |
| anti-phospho-p65 | Cell Signaling Technology | 3033S | WB, 1:1000; IF, 1:150 |
| anti-p65 | Cell Signaling Technology | 4764S | WB, 1:1000; IF, 1:200 |
| FITC anti-Mouse F4/80 | Invitrogen | 53-4801-82 | IF, 1:150 |
| anti-β-actin | Affinity | AF7018 | WB, 1:3000 |
| Goat anti-Rabbit IgG | ZSGB-BIO | ZB-2301 | WB, 1:5000 |
| Goat anti-Mouse IgG | Proteintech | SA00001-1 | WB, 1:5000 |
| Alexa Fluor 594 goat anti-rabbit IgG | Invitrogen | 2119134 | IF, 1:200 |

WB: western blot, IF: immunofluorescence
